# Recurrent volar dislocation of the metacarpophalangeal joint of the thumb with radial collateral ligament injury: A case report

**DOI:** 10.1016/j.ijscr.2020.01.056

**Published:** 2020-02-06

**Authors:** Masanori Nakayama, Yu Sakuma, Haruki Tobimatsu

**Affiliations:** a Department of Orthopaedic Surgery, School of Medicine, International University of Health and Welfare (IUHW), Mita Hospital, 1-4-3, Mita, Minato-ku, Tokyo, 108-8329, Japan; b Department of Orthopedic Surgery, Tokyo Women’s Medical University, Japan

**Keywords:** Metacarpophalangeal joint of the thumb, Open reduction, Revision surgery, Subluxation, Volar dislocation

## Abstract

**Introduction:**

A volar dislocation of the metacarpophalangeal (MCP) joint of the thumb is a rare trauma, and in combination with a radial collateral ligament (RCL) injury is much rarer. We present a surgical case with a recurrent volar dislocation of the MCP joint of the thumb with RCL injury.

**Presentation of case:**

A 47-year-old man was referred to our hospital in the subacute phase. Open reduction was performed through a dorsal incision and the RCL was repaired. X-rays taken six weeks later revealed a recurrent dislocation of the MCP joint. At the revision surgery, the extensor pollicis brevis (EPB) was detached from the proximal phalanx. As there was volar tightness, the volar plate was incised horizontally and the EPB was attached to the proximal phalanx. The final X-rays six months post-operatively revealed that the MCP joint was slightly subluxated but there was no pain on motion.

**Discussion:**

This case revealed that it is not enough only to repair the RCL to reduce a volar dislocation of the MCP joint of the thumb with an RCL injury. It revealed that re-attachment of the extensor tendons and the volar procedure are also important for a perfect reduction of a recurrent volar dislocation of the MCP joint of the thumb.

**Conclusion:**

For a volar dislocation of the MCP joint of the thumb with RCL injury, it is important not only to repair the RCL, but also to perform arthroplasty with the extensor tendons and a volar procedure to prevent recurrent dislocation after surgery.

## Highlights

• A volar dislocation of the metacarpophalangeal (MCP) joint of the thumb with radial collateral ligament (RCL) injury is rare trauma.
• A volar dislocation of the MCP joint of the thumb with RCL injury is unstable and surgical treatment is needed.
• Not only RCL repair but arthrorisis with extensor tendons is important for prevention of recurrent dislocation after surgery.

## 1 Introduction

Dorsal dislocations of the metacarpophalangeal (MCP) and carpometacarpal joint of the thumb are more common than volar dislocations [1,2]. There are limited reports of volar dislocations of the MCP joint of the thumb. Many different structures have been identified as a possible block to closed reduction, including an entrapped volar plate, dorsal capsule, collateral ligaments, or interposition of the extensor tendons [3]. Therefore, open reduction is the most common treatment for this type of dislocation because soft tissue interposition interferes with closed reduction in most cases [4,5]. Replacement and repair of interposed tissues has been recommended in most previous reports for joint reduction. There have been no reports of recurrent dislocation of the MCP joint after surgical repair.

The aim of this case report is to present a rare case of a recurrent volar dislocation of the MCP joint of the thumb with radial collateral ligament (RCL) injury treated by revision surgery. This work has been reported in line with the SCARE criteria [6].

## 2 Presentation of case

**Fig. 1:**
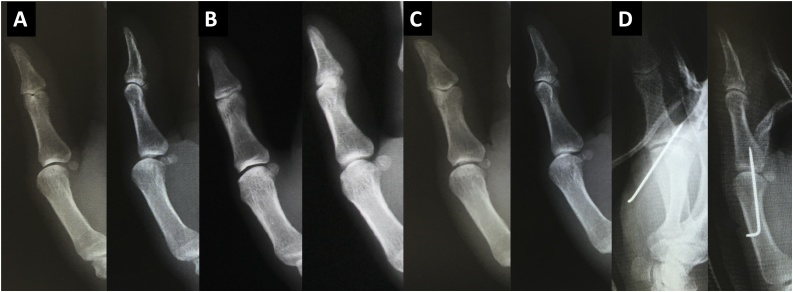
(A) Our first examination showed a volar dislocation of the metacarpophalangeal (MCP) joint. (B) After the first closed reduction, the MCP joint was reduced. (C) The MCP joint was dislocated 2 weeks after the closed reduction. (D) Immediately after the primary surgery, the MCP joint was reduced again.

A 47-year-old right-hand-dominant man had his right hand caught in a machine during his work. He presented to another clinic and the doctor treated the injury with bandage fixation to the affected thumb, and referred the patient to a hand surgeon within a few days. The patient never presented to other hospitals, and came to our hospital after about five weeks with continuous pain and lack of movement of his thumb. X-rays were taken and showed a volar dislocation of the MCP joint of the thumb (Fig. 1). A closed reduction was tried and the dislocation was reduced. The patient’s thumb was immobilized with a splint. After another two weeks, X-rays revealed a volar dislocation of the thumb MCP joint again. A closed reduction was tried once more, but the MCP joint could not be moved. Moreover, there was likely a contracture of the IP joint of his thumb as well. Surgical repair was indicated, and surgery was performed one week later.

**Fig. 2:**
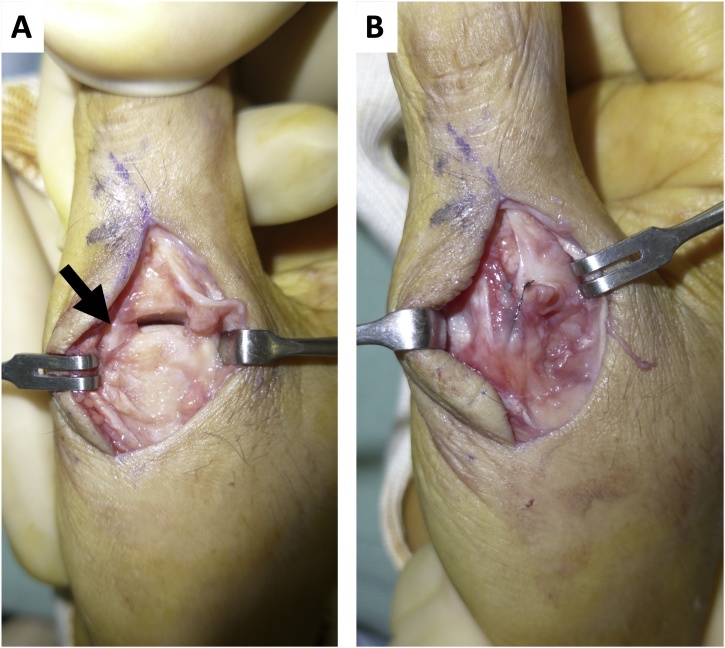
Our first surgery findings: (A) The radial collateral ligament (RCL, arrow) was interposed to the MCP joint. (B) The RCL and capsule were repaired and the MCP joint was reduced.

**Fig. 3:**
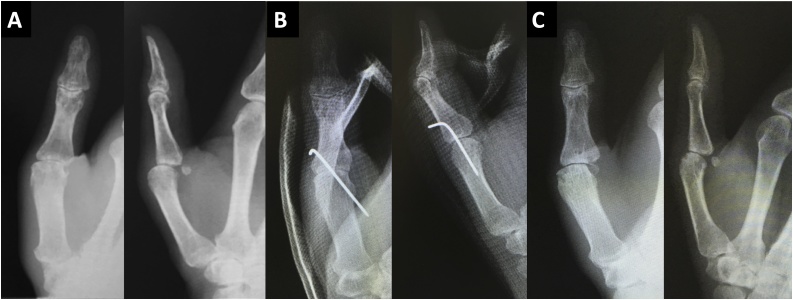
(A) Six weeks after the primary surgery, the MCP joint dislocated again. (B) Immediately after the revision surgery, the MCP joint was reduced. (C) Six months after the revision surgery, the MCP joint was slightly subluxated but not dislocated.

**Fig. 4:**
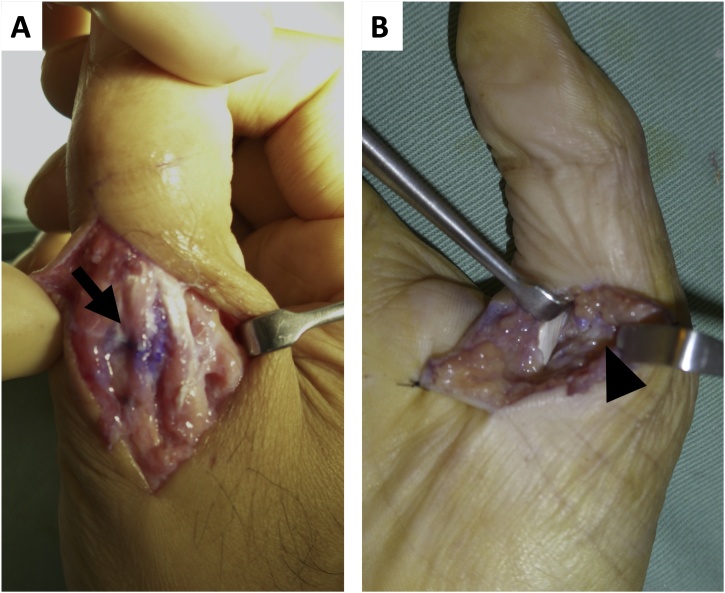
Observations on revision surgery: (A) Dorsal skin incision: the extensor pollicis brevis (EPB, arrow) was detached from the proximal phalanx of the thumb and was floating. (B) Volar skin incision: the volar plate (arrow head) was intact but slightly tight.

Under general anesthesia, a skin incision was made on the dorsal side of the MCP joint (Fig. 2). The RCL was detached from the proximal phalanx of the thumb and interposed to the MCP joint. The capsule was partly ruptured but the ulnar collateral ligament (UCL) and volar plate appeared intact. The extensor pollicis longus (EPL) and extensor pollicis brevis (EPB) appeared intact and were not obstacles to the dislocated joint. The RCL was reduced and attached to the proximal phalanx using a suture to anchor it, and the capsule was repaired. Fluoroscopy showed the MCP joint was returned to a good position and temporary fixation of the MCP joint was accomplished using Kirschner wire (Fig. 1). The thumb was immobilized postoperatively with a thumb spica splint for four weeks and then the wire was removed and the patient was allowed to move the thumb. Six weeks after the surgery, the patient complained that the MCP and IP joints were painful on motion and their motion was limited. X-rays revealed recurrent dislocation of the MCP joint (Fig. 3). Revision surgery under general anesthesia was performed one week later. Surgical observations revealed that the repaired RCL was not ruptured or elongated, but the EPB was dislocated from its original position, and the EPL was adherent to other tissues, which restricted its movement (Fig. 4). As there was volar tightness, a volar skin incision was made and it was found that the volar plate was intact but slightly tight (Fig. 4). Since the tight volar plate seemed to interrupt a reduction of the joint, a horizontal incision was made on the volar plate. The EPB was attached to the proximal phalanx using a suture as an anchor, and EPL adhesion to other tissues was released. Fluoroscopy showed the MCP joint was returned to a good position. The MCP joint was fixed temporarily with a wire for six weeks (Fig. 3) and then removed. Six months after revision surgery, X-rays revealed the MCP joint was slightly subluxated (Fig. 3), but was stable and congruent. Extension and flexion of the MCP joint were −10° and 50°, and those of the IP joint were 0° and 70°. There were no complaints of pain or limitations of activities, and the patient returned to his original job.

## 3 Discussion

A volar dislocation of the MCP joint of the thumb is a rare trauma. There have been fewer than thirty cases reported in the English literature [[1], [2], [3], [4], [5],^7^]. Moreover, there are no reports of recurrent dislocation of the MCP joint after surgical repair. Although the maximum follow-up period reported in the literature was 36 months, most studies report follow-up periods from three to six months, similar to this case. Common surgical findings of this type of dislocation are metacarpal head herniation through the tear of the dorsal capsule, EPL and EPB tendon dislocation, and rupture of the UCL and volar plate [8]. Gunter and Zielinski reported a case of irreducible volar dislocation of the MCP joint of the thumb in which EPB and EPL tendons were trapped beneath the metacarpal head [9]. In this case, we confirmed a tear of the dorsal capsule with a ruptured RCL at the first surgery, but EPB and EPL looked to be intact. An atypical aspect of this case was that the RCL injury was associated with the dislocation, whereas many of the previous reports showed that UCL injury was associated with the volar dislocation. Some authors have reported that acute or chronic RCL injury might be the cause for MCP joint subluxation [5,10,11], and that repairing the collateral ligaments results in stabilizing the joint [5,7]. Other authors have reported that a thumb MCP volar dislocation can be treated surgically with a dorsal incision only [4,8]. Considering the current case, in which the thumb MCP joint dislocated with an RCL injury, it might be desirable during the first surgery not only to repair the RCL but also to attach the EPL and/or EPB to the proximal phalanx, even if they appear to be intact. Furthermore, if a dorsal incision procedure cannot reduce the joint perfectly, or a recurrent joint dislocation occurs after surgery with the dorsal incision only, a volar skin incision and treatment of the volar plate should be done. In previous reports, major post-operative therapies were immobilization of the MCP joint with Kirschner wires for three or four weeks [5] or with a thumb spica splint for three to six weeks [1,2]. These approaches were similar to our method at the time of the first surgery. However, arthroplasty with the extensor tendons at the first surgery in this case should have been performed to prevent recurrent dislocation.

Senda and Okamoto described a new classification for this type of MCP joint dislocation [5]: type A, stable; type B, blocked; and type C, unstable. Most of type A cases did not require open reduction. Type B was reported to be the most common pattern associated with UCL rupture and required open reduction. Type C was associated with gross ligament injury, and early open reduction and ligament reconstruction has been recommended. However, it also was reported that recurrent joint dislocation might occur with poor outcomes related to stiffness, pain and loss of pinch strength. Although our case might be type C, it could not be confirmed because it was already in the subacute phase and the dislocated joint was tight at the patient’s first visit to our hospital. Even if the type of dislocation is unclear, it is necessary to realize the possibility of a recurrent dislocation with a volar MCP joint dislocation of the thumb, and to treat the extensor tendons in addition to the ligaments and joint capsules at the initial surgery. Also, if necessary, a volar incision and procedure should be done.

## 4 Conclusion

As the literature regarding recurrent volar dislocation of the MCP joint of the thumb is limited, its treatment is difficult and should be considered very carefully. Especially in Senda’s type C dislocation, surgical exploration and repair of the extensor tendons in addition to the ligament and capsule is needed. If a dorsal incision procedure cannot reduce the joint perfectly or a recurrent joint dislocation occurs after surgery, a volar incision to observe the volar plate and achieve a perfect reduction of the dislocation may be necessary.

## Sources of funding

This study has not received any external funding.

## Ethical approval

Since our article is a case report, no approval from the Ethics Committee is required in our institution.

## Consent

Written informed consent was obtained from the patient for publication of this case report and accompanying images. A copy of the written consent is available for review by the Editor-in-Chief of this journal on request.

## Author contribution

Masanori Nakayama is a main surgeon of this case, conceptualized and drafted the manuscpript.

Yu Sakuma helped to treat this case and edited the manuscpript.

Haruki Tobimatsu helped to treat this case.

## Registration of research studies

Not applicable.

## Guarantor

Masanori Nakayama.

## Provenance and peer review

Not commissioned, externally peer-reviewed.

## Declaration of Competing Interest

The authors declare that they have no conflicts of interest.

## References

[1] Yüksel S., Adanır O., Beytemur O., Gülec A.M. (2017). Volar dislocation of the metacarpophalangeal joint of the thumb: a case report. Acta Orthop. Traumatol. Turc..

[2] Potini V.C., Sood A., Sood A., Mastromonaco E. (2014). Volar dislocation of the thumb metacarpophalangeal joint with acute repair of the ulnar collateral ligament. Case Rep. Plast. Surg. Hand Surg..

[3] Moneim M.S. (1983). Volar dislocation of the metacarpophalangeal joint. Pathologic anatomy and report of two cases. Clin. Orthop..

[4] Miyamoto M., Hirayama T., Uchida M. (1986). Volar dislocation of the metacarpophalangeal joint of the thumb: a case report. J. Hand Surg..

[5] Senda H., Okamoto H. (2014). Palmar dislocation of the thumb metacarpophalangeal joint: report of four cases and a review of the literature. J. Hand. Surg. Eur..

[6] Agha R.A., Borrelli M.R., Farwana R., Koshy K., Fowler A., Orgill D.P., SCARE Group (2018). The SCARE 2018 statement: updating consensus Surgical CAse REport (SCARE) guidelines. Int. J. Surg..

[7] Garcia M.S., Hidalgo O.A., Martinez G.M. (1991). Volar dislocation of the first metacarpophalangeal joint. A case report and review of the literature. Acta Orthop. Belg..

[8] Singhal R.K. (1974). Anteromedial dislocation of metacarpophalangeal joint of the thumb with special emphasis on its mechanism of injury. J. Indian Med. Assoc..

[9] Gunther S.F., Zielinski C.J. (1982). Irreducible palmar dislocation of the proximal phalanx of the thumb. A case report. J. Hand Surg..

[10] Edelstein D.M., Kardashian G., Lee S.K. (2008). Radial collateral ligament injuries of the thumb. J. Hand Surg..

[11] Taylor K.F., Lanzi J.T., Cage J.M., Drake M.L. (2013). Radial collateral ligament injuries of the thumb metacarpophalangeal joint: epidemiology in a military population. J. Hand Surg..

